# Metavirome Analysis of Viruses Carried by Dairy Cows in Shaanxi, Gansu and Ningxia, China

**DOI:** 10.3390/ani16121928

**Published:** 2026-06-22

**Authors:** Yanling Liu, Gang Zhang, Hui Gao, Min Fang, Lingling Jiang, Yongyi Kong, Qiang Liu, Pu Wang, Sinong Zhang, Yong Li

**Affiliations:** 1 College of Life Sciences, Ningxia University, Yinchuan 750021, China; lyl315176@163.com (Y.L.); fuzang123@163.com (G.Z.); 15121945891@163.com (H.G.); 13895473421@163.com (M.F.); jianglingling0512@163.com (L.J.); qfkongyy@163.com (Y.K.); liuqiang125210@163.com (Q.L.); wangpu1175@163.com (P.W.); 2 Key Laboratory of Conservation and Utilization of Biological Resources in Western China, Ministry of Education, Ningxia University, Yinchuan 750021, China

**Keywords:** dairy cows, metavirome, Shaanxi–Gansu–Ningxia region, virus diversity, phylogenetic analysis

## Abstract

Dairy cows are economically significant ruminants in China, and the dairy industry is closely linked to food safety and the agricultural economy. However, various factors such as pathogenic microorganisms often lead to frequent diseases in dairy cows. Furthermore, as potential hosts for diverse viruses, dairy cows can harbor zoonotic pathogens, which pose a threat to public health. The Shaanxi–Gansu–Ningxia region boasts abundant natural resources and extensive pastures. It is a major animal husbandry base in Northwest China, and dairy farming plays a significant role in the local economy. However, research on dairy cow virus diversity in this region remains limited; epidemic prevention and control capabilities are constrained, and the risk of disease outbreaks is elevated. In this study, 790 dairy cow samples were collected from 13 large-scale farms and free-range households in the Shaanxi–Gansu–Ningxia region from 2021 to 2023. Sample types consisted of nasal and anal swabs. Six viral metagenomic libraries were constructed and analyzed using high-throughput sequencing and bioinformatics methods, leading to the identification of 51 viral families. These comprised 16 positive-sense single-stranded RNA virus families, one *Retroviridae* family, four double-stranded RNA virus families, 21 double-stranded DNA virus families, and nine single-stranded DNA virus families. Among these, RNA viruses were represented by families such as *Astroviridae*, *Coronaviridae*, *Caliciviridae*, *Picornaviridae*, and *Picobirnaviridae*; DNA viruses were primarily detected in *Circoviridae*, *Papillomaviridae*, *Genomoviridae*, and *Smacoviridae*. Alpha diversity analysis revealed no significant differences in viral diversity and abundance among the three regions (*p* > 0.05); however, significant differences were observed in the read counts and proportions of RNA and DNA viruses across the provinces. Phylogenetic analysis further indicated that viruses carried by dairy cows exhibit considerable genetic diversity and pose potential cross-species transmission risks. This study established a reference database for the dairy cow virome in the Shaanxi–Gansu–Ningxia region, elucidated the phylogenetic relationships of key viruses, and provided a scientific basis for future monitoring and prevention of dairy cow viruses.

## Simple Summary

To address the knowledge gap regarding viral diversity and the high risk of infectious diseases in dairy cattle in the Shaanxi–Gansu–Ningxia region of northwestern China, this study collected 790 samples from 2021 to 2023 and systematically profiled the viral composition carried by dairy cattle in this region using metagenomic sequencing. A total of 51 viral families were identified, encompassing various RNA and DNA viruses, which exhibited high genetic diversity and significant potential for cross-species transmission. Although no significant differences were observed in overall viral diversity across regions, substantial variations in the abundance and proportion of RNA and DNA viral reads were detected among the three provinces. This study established the first reference virome database for dairy cattle in the Shaanxi–Gansu–Ningxia region, providing a crucial scientific foundation for disease surveillance, control strategies, and public health risk early warning systems.

## 1. Introduction

As one of the most important livestock species globally, dairy cows are crucial for milk production. With the growing global demand for dairy products, dairy farming has become a crucial component of modern agricultural systems. In China, dairy cows not only serve as the primary source of milk but also constitute a vital pillar industry for promoting rural economic development [1]. However, the health and productivity of dairy cows are consistently threatened by various viral pathogens. Dairy cows harbor a highly diverse array of viruses spanning multiple families. Epidemiological studies have revealed that bovine viral diarrhea virus (BVDV) exhibits a multi-genotype epidemic pattern in China. Serological data indicate a national infection rate of 53%, with seropositivity rates as high as 90% in Fujian Province [2]. Notably, an outbreak of foot-and-mouth disease type Asia 1 was first reported on dairy farms in Shandong and Jiangsu in 2005, subsequently leading to transmission chains across 10 provinces and regions and resulting in substantial economic losses [3]. Among these pathogens, bovine coronavirus (BCoV) is a major causative agent of calf diarrhea, winter dysentery, and bovine respiratory disease complex. Its national prevalence is 30.8%, with a significantly higher (60.5%) detection rate in southern China than in other regions [4]. In terms of emerging infectious diseases, the lumpy skin disease outbreak reported in Yili, Xinjiang, in 2019 was caused by the highly pathogenic lumpy skin disease virus. Owing to its high transmissibility, the virus spread to 14 provinces within two years and has since been included in the list of Category I notifiable animal diseases requiring quarantine in China [5]. Furthermore, dairy cows can carry and transmit zoonotic pathogens, which not only threaten their health but may also be transmitted to humans through direct contact or the food chain. Common zoonotic pathogens include viruses such as Rift Valley fever virus [6], rabies virus [7], bovine influenza A virus [8] and bovine Japanese encephalitis virus [9], as well as bacteria such as *Salmonella* spp. and *Brucella abortus* [10]. Therefore, comprehensive surveillance and identification of viruses in dairy cattle and their environment are essential for obtaining accurate monitoring data and predicting epidemic risks.

Metavirome analysis is a powerful tool for the comprehensive profiling of viral communities in environmental and host samples [11]. Based on next-generation sequencing (NGS) technology, this approach characterizes all viral genetic material in a sample to offer clues regarding the species diversity, genetic evolution, and potential functional profiles of viral communities [12]. This technology has been successfully applied to identify viruses in animal hosts such as pigs, dogs, and poultry. It has not only uncovered numerous novel viral genomes but also enabled the tracking of their evolutionary dynamics. For instance, the analysis of fecal samples from diarrheal piglets in Jiangsu Province, China, revealed multiple novel viral genotypes, whose community variations were significantly correlated with farming environments and management practices [13]. Analysis of the digestive tract virome of plateau dogs reveals patterns of cross-species transmission and identifies novel viral lineages with potential zoonotic risk [14]. Furthermore, systematic monitoring of live poultry markets in Guangdong using metaviromics provided crucial data on the dynamics of virus species and their abundance [15]. These studies demonstrate the significant value of metaviromics in virus discovery and surveillance.

The Shaanxi–Gansu–Ningxia region, located in northwestern China, comprises three geographically contiguous provinces that form a distinct ecological and economic entity. It features diverse geographical conditions, including mountains, grasslands, and deserts, with climates ranging from semi-arid to temperate continental. The region possesses relatively abundant water and agricultural resources, making it a key area for livestock breeding in China. Dairy farming is a vital component of the local economy; however, fundamental research on cattle diseases remains limited. The prevalence and genetic variation in viruses are poorly characterized, and data on viral diversity are scarce. Moreover, the region faces threats from emerging and imported infectious diseases, which pose significant risks to public health. Therefore, conducting metavirome studies in the Shaanxi–Gansu–Ningxia region is essential to elucidate the diversity, abundance, and dynamics of viruses in dairy cows. This research will provide a scientific basis for the detection, prevention, and control of viral diseases in the region’s dairy cattle.

## 2. Materials and Methods

### 2.1. Sample Collection and Preparation

**Figure 1:**
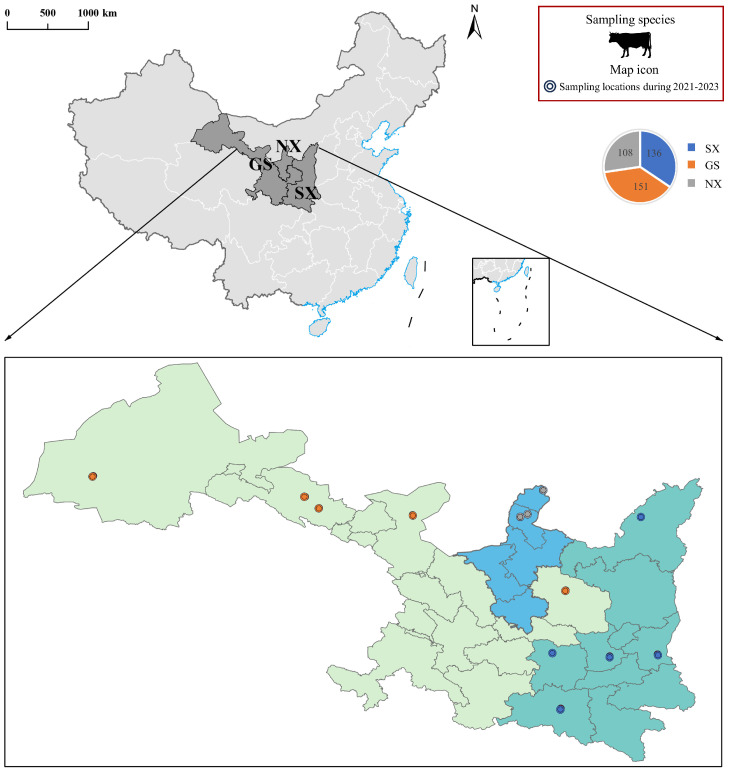
Distribution of dairy cattle sampling sites in Shaanxi, Gansu and Ningxia regions: The circular markers in the map indicate the locations of dairy cow sampling sites in 13 prefecture-level cities in the Shaanxi, Gansu and Ningxia regions (GS: Gansu Province, NX: Ningxia Hui Autonomous Region, SX: Shaanxi Province). The pie chart shows the number of cows collected in the three regions. The maps are based on standard maps downloaded from the National Geographic Information Public Service Platform (NGIPSP) with review number GS(2024)0650.

From December 2021 to June 2023, stratified random sampling was used to select large-scale dairy farms and free-range households across 13 counties in Shaanxi, Gansu and Ningxia. Sampling sites were distributed across prefecture-level cities to avoid clustering bias (Figure 1). Tested dairy cows were selected via cluster random sampling within each farm, with a total of 395 animals enrolled. One nasal swab and one anal swab were collected simultaneously from each cow, yielding 790 samples in total, including 272 from Shaanxi, 302 from Gansu and 216 from Ningxia. Detailed geographical information of the sampling sites is presented in Figure 1. All samples were collected by licensed veterinarians. Prior to sampling, on-site clinical examination was performed for all cattle; animals with abnormal clinical manifestations or a previous disease history were excluded from sampling. All samples were collected using disposable materials, transported to the laboratory in liquid nitrogen, and subsequently stored at −80 °C. For sample processing, each swab was vigorously vortexed in a virus preservation solution for 10 min, followed by centrifugation at 12,000 rpm for 10 min at 4 °C; the resulting supernatant was then collected for subsequent analysis. The purpose and procedures of this study were fully explained to all cattle owners, and informed consent for sample collection was obtained.

### 2.2. Nucleic Acid Extraction, Library Construction, and Sequencing

The 790 samples were divided into three pools according to their regions of origin: Shaanxi, Gansu, and Ningxia. The supernatant of each sample was filtered through a 0.45 μm filter (Millipore, Burlington, MA, USA) to remove eukaryotic cells and other large particles, and then concentrated to 2 mL via ultrafiltration centrifugation at 4000 rpm and 4 °C using a 100 kDa ultrafiltration tube (Millipore, Burlington, MA, USA). RNA was extracted from the concentrates using the Trizol method, followed by rRNA depletion with the rRNA Removal Kit (Bio-Rad, Hercules, CA, USA). DNA was extracted using a genomic DNA extraction kit (TIANGEN, Beijing, China) and subsequently amplified with the Illustra GenomiPhi V2 DNA Amplification Kit (Cytiva, Uppsala, Sweden). The quality and concentration of the extracted RNA and DNA were assessed using an Agilent 2100 Bioanalyzer (Agilent, Santa Clara, CA, USA). Qualified samples were used to construct sequencing libraries with the Illumina TruSeq library preparation protocol (Illumina, San Diego, CA, USA). Samples were grouped according to geographic origin and nucleic acid type (DNA/RNA), and six final virome libraries were constructed and designated as SXN-RNA, GSN-RNA, NXN-RNA, SXN-DNA, GSN-DNA and NXN-DNA, respectively. The libraries were sequenced by Novogene Co., Ltd. (Beijing, China) using the Illumina HiSeq high-throughput sequencing platform.

### 2.3. Bioinformatics Analysis

Raw sequencing reads generated from Illumina sequencing were quality-controlled and filtered using Trimmomatic software (Trimmomatic 0.39) [16]. Briefly, Illumina adapter sequences were removed; low-quality terminal bases were trimmed, and reads shorter than 36 bp after trimming were discarded [17]. The results of multiple k-mer assemblies were evaluated within IDBA-UD to select the optimal assembly based on technical parameters, including the total length of assembled sequences, the number of contigs, and the N50 value. The assembled contigs from each library were compared against the non-redundant nucleotide (NT) and protein (NR) databases using BLAST (https://blast.ncbi.nlm.nih.gov/Blast.cgi (accessed on 15 June 2026)), with an E-value threshold set to < 1 × 10^−5^. Subsequently, the candidate viral sequences were aligned against a non-viral, non-redundant (NVNR, Non-Viral Non-Redundant) protein database to exclude false-positive hits. The NVNR database was constructed from NCBI nr FASTA files, from which all viral sequences were eliminated according to taxonomic classification and only non-viral protein sequences were reserved for false-positive filtering [11]. Finally, the taxonomic classification results from both BLASTn (https://blast.ncbi.nlm.nih.gov/Blast.cgi?PROGRAM=tblastn&PAGE_TYPE=BlastSearch&LINK_LOC=blasthome (accessed on 15 June 2026)) [18] and BLASTx (https://blast.ncbi.nlm.nih.gov/Blast.cgi?PROGRAM=blastx&PAGE_TYPE=BlastSearch&LINK_LOC=blasthome (accessed on 15 June 2026)) [18] were parsed using MEGAN (11) [19], which employs the lowest common ancestor (LCA) algorithm.

### 2.4. Diversity, Abundance, Shared and Specificity Analysis of Viruses

Reads from all six libraries were annotated to specific viral families and genera, with the major viral sequences being verified by BLASTx. To visualize the viral composition and abundance within the libraries, a heatmap was generated using ChiPlot (https://www.chiplot.online/). To comprehensively analyze the viral alpha diversity across the six libraries, richness was characterized based on viral families and genera, while the Shannon [20] and Richness [21] indices were employed to reflect diversity and abundance, respectively. These indices were calculated using the vegan package in RStudio (2023.09.0) [22], and the results were visualized with ChiPlot. A Venn diagram was constructed to illustrate the shared and unique viruses among the three provinces.

### 2.5. Phylogenetic Analysis

Viral nucleotide sequences obtained in this study, viral sequences with the highest matches obtained through BLASTx searches of the GenBank database, and representative members of related viral species or genera were used for phylogenetic analysis. Phylogenetic trees were constructed based on Capsid protein, RdRp (RNA-dependent RNA polymerase), and other protein sequences. First, multiple sequence comparisons were performed in MEGA 11 [23] using the Clustal W algorithm [24] with default settings. Subsequently, phylogenetic analyses were implemented in MEGA 11. Maximum Likelihood (ML) was selected as the primary phylogenetic construction strategy, while Neighbor-Joining (NJ) was adopted as an auxiliary verification approach. To ensure result robustness, all target sequences were independently analyzed by both algorithms, and cross-comparison confirmed identical topological structures for all trees. [25] Bootstrap tests with 1000 replicates were conducted to evaluate tree confidence. Finally, the phylogenetic trees were annotated and trimmed using Adobe Illustrator 2022 v.26.0.1 and ChiPlot.

## 3. Results

### 3.1. Overview of the Metavirome

To characterize viral communities hosted by dairy cows across Shaanxi, Gansu and Ningxia, nasal and anal swabs were collected from 395 clinically healthy dairy cows covering 13 counties between December 2021 and June 2023 (Figure 1). All six constructed libraries were subjected to Illumina HiSeq high-throughput sequencing, and detailed sequencing data for each library are summarized in Table 1.

The sequencing results revealed that among the RNA libraries, the SXN-RNA library contained 69,773,276 clean reads, including 23,475 viral reads (0.03%); the GSN-RNA library contained 73,017,634 clean reads, with 96,984 viral reads (0.13%); and the NXN-RNA library contained 51,775,112 clean reads, including 98,690 viral reads (0.19%). Among the DNA libraries, the SXN-DNA library had 69,388,876 clean reads and 8,269,370 viral reads (11.92%); the GSN-DNA library had 69,294,598 clean reads and 3,543,148 viral reads (5.11%); and the NXN-DNA library contained 73,510,804 clean reads and 1,544,101 viral reads (2.10%) (Table 1).

### 3.2. Composition and Comparison of Viral Communities

**Figure 2:**
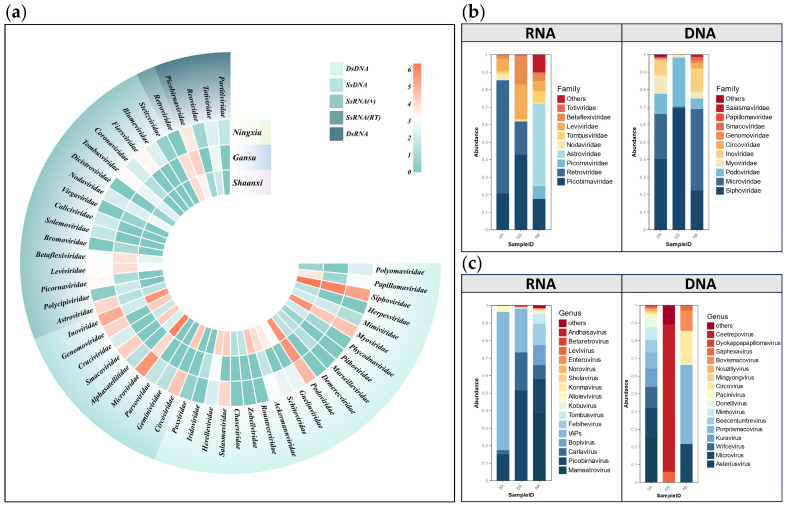
Statistical analysis of virus communities in dairy cattle in Shaanxi, Gansu and Ningxia regions: (**a**) Heatmap showing the abundance distribution of different virus families in the three regions of Shaanxi, Gansu and Ningxia. The abundance data were log10 transformed, and the sampling regions, nucleic acid types and virus families were labeled using different colors (see color legend for details); (**b**) Proportion of relative abundance based on virus family level classification; (**c**) Proportion of relative abundance based on virus genus level classification.

In order to demonstrate the differences in the composition of viral communities in the three regions of Shaanxi, Gansu and Ningxia, a heat map was constructed for analysis (Figure 2a). At the family level, 51 virus families were identified, including 16 single-stranded positive-sense RNA [SsRNA(+)] virus families, one single-stranded RNA reverse transcription [SsRNA(RT)] virus family, four double-stranded RNA (DsRNA) virus families, 21 double-stranded DNA (DsDNA) virus families, and nine single-stranded DNA (SsDNA) virus families.

At the family/genus level (Figure 2b,c), viruses of the genus *Picobirnavirus* (family *Picobirnaviridae*) were ubiquitously detected across all sampled libraries, with their relative abundance exceeding 50% in the Gansu region. Similarly, viruses of the genus *Mamastrovirus* (family *Astroviridae*) were present in all libraries, showing the highest relative abundance in the Ningxia region. The family *Picornaviridae* also demonstrated high relative abundance in Ningxia. Subsequent classification of the reads assigned them to several genera, including *Enterovirus*, *Bopivirus*, *Aphthovirus*, and *Kobuvirus*.

Notably, the Ningxia region exhibited a greater number of genera with high relative abundance of RNA viruses compared to the Shaanxi and Gansu regions. Specifically, the high-abundance genera in Shaanxi were exclusively limited to *Picobirnavirus* (*Picobirnaviridae*) and viruses of the family *Retroviridae*. In contrast, the RNA viral community in Gansu was predominantly composed of *Picobirnavirus* (*Picobirnaviridae*) and *Carlavirus* (*Betaflexiviridae*). Regarding DNA viruses, the community was primarily composed of bacteriophages, followed by CRESS DNA viruses. The latter were mainly classified into the genera *Circovirus* (family *Circoviridae*), *Bovismacovirus*, and *Porprismacovirus* (both of the family *Smacoviridae*). Regional distribution analysis revealed distinct patterns. Shaanxi harbored a relatively high number of high-abundance DNA virus genera. In contrast, the high-abundance genera in Gansu were exclusively limited to *Ceetrepovirus* (family *Potyviridae*) and *Saphexavirus* (family *Siphoviridae*). Conversely, the DNA viral community in Ningxia was primarily dominated by *Bovismacovirus* and *Porprismacovirus* (*Smacoviridae*), *Circovirus* (*Circoviridae*), and *Microvirus* (*Microviridae*).

**Figure 3:**
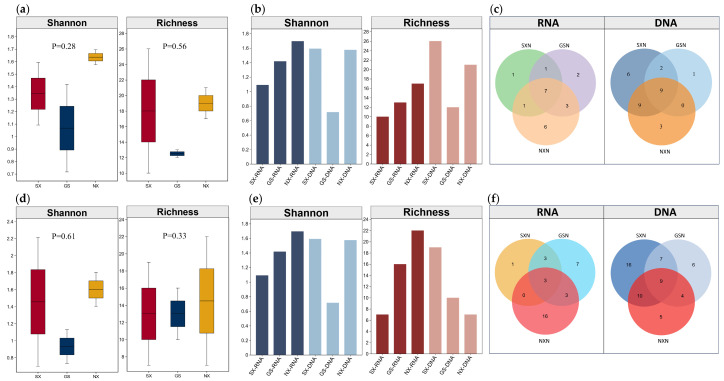
Statistical analysis of virus communities in dairy cattle in Shaanxi, Gansu and Ningxia regions: (**a**,**d**) Box line plot of Alpha diversity index of cow viruses in Shaanxi, Gansu and Ningxia based on family/genus level classification ((**a**) family (**d**) genus); (**b**,**e**) Alpha diversity index in the dairy cow library based on virus family/genus level classification ((**b**) family (**e**) genus); (**c**,**f**) Distributional characteristics of virus families/genera in Shaanxi, Gansu and Ningxia provinces: comparison of shared and endemic virus families/genera ((**c**) family (**f**) genus).

To further explore the differences among viral communities, the Shannon and Richness indices were employed to assess viral alpha diversity and abundance. At both the viral family and genus levels, no significant differences were observed in the overall RNA and DNA viral diversity and abundance among the three provinces (*p* > 0.05); however, significant differences were found in the read counts and proportions of RNA and DNA viruses across the provinces (Figure 3a,d). Notably, the Ningxia region exhibited the highest alpha diversity and abundance of RNA viruses, whereas the Shaanxi region showed the highest alpha diversity and abundance of DNA viruses (Figure 3b,e).

The distribution of RNA and DNA virus families/genera in Shaanxi, Gansu and Ningxia provinces was characterized as shown in (Figure 3c,f). At the family level, seven RNA viral families and nine DNA viral families were shared across all three regions. Notably, several region-specific viral families were identified: *Caliciviridae* and *Parvoviridae* in Ningxia; *Herpesviridae* in Shaanxi; and *Iridoviridae* in Gansu. At the genus level, three RNA viral genera and nine DNA viral genera were common to all provinces. Important region-specific genera included *Orthoreovirus* in Shaanxi; *Betacoronavirus* and *Gammaretrovirus* in Gansu; and *Aphthovirus*, Norovirus, and *Kobuvirus* in Ningxia.

### 3.3. Analysis of the Genetic Evolution of Dairy Cattle Viruses

#### 3.3.1. *Astroviridae*

**Figure 4:**
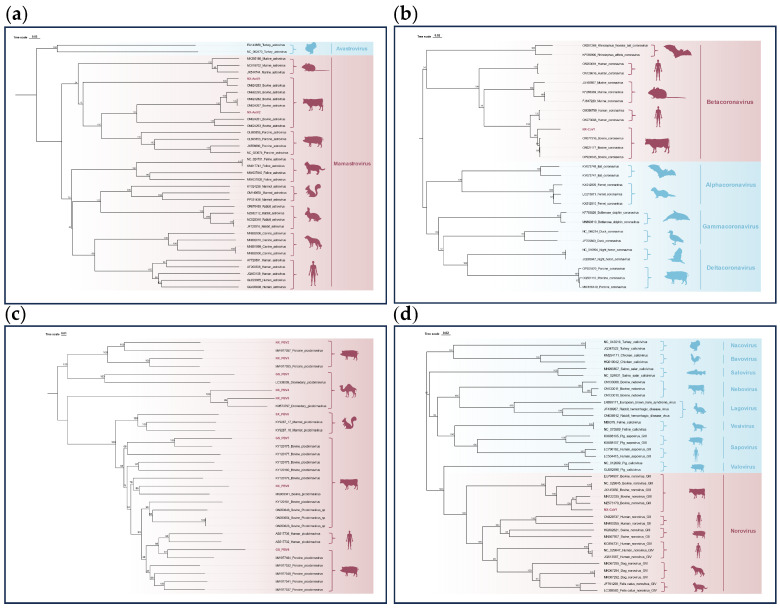
Phylogenetic analysis of relationships of RNA viruses: (**a**) Phylogenetic tree constructed based on the nucleotide sequences of the Capsid gene from the family *Astroviridae*; (**b**) Phylogenetic tree built from the nucleotide sequences of the ORF1ab gene of the family *Coronaviridae*; (**c**) Phylogenetic tree established using the nucleotide sequences of the RdRp gene of the family Picobirnaviridae; (**d**) Phylogenetic tree inferred from the nucleotide sequences of the ORF1 gene of the family Caliciviridae. Sequences highlighted in red represent those identified in the present study.

Astroviruses are positive-sense single-stranded RNA viruses with a genome length of approximately 6.2–7.7 kb, characterized by three overlapping open reading frames (ORFs) [26]. The family *Astroviridae* comprises two genera, *Mamastrovirus* and *Avastrovirus*, which primarily infect mammalian and avian hosts, respectively [27]. In this study, two nearly complete astrovirus capsid gene nucleotide sequences (2199 bp and 2250 bp in length) were successfully obtained from the Ningxia RNA libraries. Phylogenetic analysis revealed that both novel sequences clustered within the *Mamastrovirus* genus (Figure 4a). Sequence alignment indicated that NX-AstV1 shared 96.15% nucleotide identity with a bovine astrovirus strain from China (GenBank: ON624283), while NX-AstV2 showed 92.86% identity with another Chinese bovine astrovirus strain (GenBank: ON682290). These findings suggest a close evolutionary relationship between the newly identified astrovirus sequences from the Ningxia region and existing bovine astrovirus strains in China.

#### 3.3.2. *Coronaviridae*

Coronaviruses are enveloped, positive-sense single-stranded RNA viruses with genomes ranging from 26 to 33 kb in size [28]. Studies have confirmed that these viruses can cause respiratory, gastrointestinal, and neurological diseases in a wide range of mammalian hosts, including humans [29]. In 2019, Coronavirus Disease 2019 (COVID-19) erupted into a global pandemic, with cumulative confirmed cases exceeding hundreds of millions to date [30]. Given their demonstrated capacity for cross-species transmission and potential to trigger significant public health emergencies, the continuous monitoring and in-depth research of their animal hosts (e.g., bats, rodents) are of paramount public health importance. In this study, a coronavirus ORF1ab gene sequence (1031 bp in length) was successfully obtained. Phylogenetic analysis revealed that it clusters within the genus *Betacoronavirus* (Figure 4b), which encompasses several pathogens of major public health concern, including Severe Acute Respiratory Syndrome Coronavirus (SARS-CoV), Middle East Respiratory Syndrome Coronavirus (MERS-CoV), and Severe Acute Respiratory Syndrome Coronavirus 2 (SARS-CoV-2) [31]. Notably, NX-CoV1 exhibits 95.69% nucleotide identity with a bovine coronavirus from China (GenBank accession no. OR077316). It also shows 95.41% and 95.11% identity with human coronavirus OC43 strains detected in Canada (OM386799) and Hebei, China (OK073088), respectively. Such high genetic similarity indicates that NX-CoV1 may share a common evolutionary origin with the above strains or suggests a close evolutionary relationship between them.

#### 3.3.3. *Picobirnaviridae*

Picobirnavirus is a small, non-enveloped double-stranded RNA virus with a genome consisting of two segments: the larger segment encodes a polyprotein (ORF1) and a capsid protein (ORF2), while the smaller segment encodes the RNA-dependent RNA polymerase (RdRp) [32]. This virus primarily infects vertebrate hosts and has been widely detected in fecal samples and intestinal environments of both animals and humans [33]. In this study, nine sequences of the RdRp gene from picobirnaviruses (ranging from 618 to 1697 bp in length) were obtained from the Shaanxi, Gansu, and Ningxia regions of China. A phylogenetic tree constructed based on the nucleotide sequences of the RdRp gene revealed that these novel sequences form distinct clades with picobirnavirus strains of bovine (*Bovine picobirnavirus*), dromedary camel (*Camelus dromedarius picobirnavirus*), porcine (*Porcine picobirnavirus*), and marmot (*Marmota picobirnavirus*) origins (Figure 4c). Nucleotide sequence similarity analysis showed that GS-PBV7 and NX-PBV8 share 77.82–85.77% similarity with bovine strains; GS-PBV1, NX-PBV4, and NX-PBV5 exhibit 78.52–93.24% similarity with dromedary camel strains; NX-PBV2, NX-PBV3, and GS-PBV9 demonstrate 80.74–88.57% similarity with porcine strains; and SX-PBV6 shares 75.96% similarity with a marmot strain (GenBank accession no. KY928717).

#### 3.3.4. *Caliciviridae*

The family *Caliciviridae*, comprising 11 recognized genera including *Norovirus*, *Nebovirus*, *Sapovirus*, *Vesivirus*, *Salovirus*, *Valovirus*, *Lagovirus*, *Minovirus*, *Recovirus*, *Bavovirus*, and *Nacovirus*, is known to infect a wide range of animal and human hosts [34]. Among these, *Norovirus* is primarily transmitted via the fecal–oral route and represents a major causative agent of viral gastroenteritis in humans [35]. In this study, a novel sequence of 754 bp in length was identified and classified within the genus *Norovirus*. Phylogenetic analysis based on the nucleotide sequences of the ORF1 region revealed that NX-CaV1 clusters within the same clade as *Norovirus* GIII strains detected in Henan Province (GenBank accession no. MN122335) and Hebei Province (GenBank accession no. MZ573179), China, sharing nucleotide sequence identities of 91.80% and 90.59%, respectively (Figure 4d). This high degree of sequence identity suggests that NX-CaV1 may share a common evolutionary origin or a recent common ancestor with known *Norovirus* GIII strains.

#### 3.3.5. *Picornaviridae*

**Figure 5:**
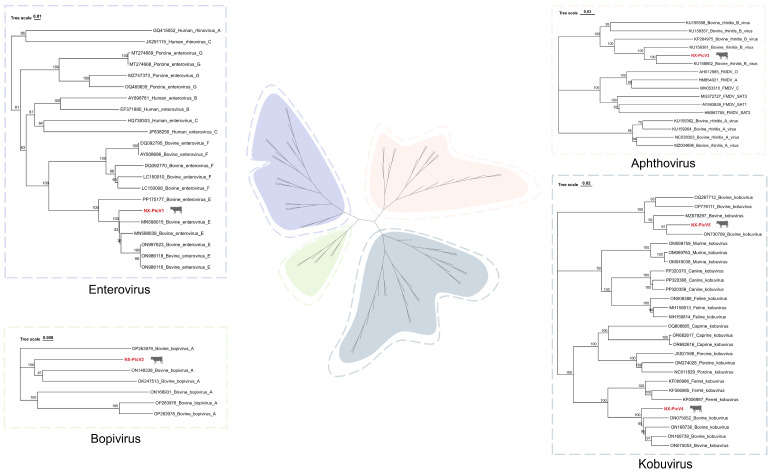
Phylogenetic analysis of relationships in the family of *Picornaviridae*: A phylogenetic tree was constructed based on the nucleotide sequences of the polyprotein gene from the family *Picornaviridae*, encompassing the genera Enterovirus, Bopivirus, Aphthovirus, and Kobuvirus. Sequences highlighted in red represent those identified in the present study.

The family *Picornaviridae* comprises small, non-enveloped, single-stranded RNA viruses that include numerous highly infectious pathogens affecting both humans and animals. These viruses can cause a wide spectrum of diseases, ranging from mild respiratory illnesses to severe conditions such as encephalitis, hepatitis, and myocarditis [36]. In this study, five novel polyprotein nucleotide sequences were obtained, belonging to the genera *Enterovirus*, *Bopivirus*, *Aphthovirus*, and *Kobuvirus*. Phylogenetic analysis revealed that NX-PicV1 shares 92.95% nucleotide identity with *Bovine enterovirus E* (GenBank accession no. ON986118); NX-PicV2 shows 86.39% identity with *Bovine bopivirus A* (ON148339); NX-PicV3 exhibits 85.45% identity with *Bovine rhinitis B virus* (KU168862); and NX-PicV4 and NX-PicV5 demonstrate 89.87% and 91.40% identity with *Bovine kobuvirus*, respectively (Figure 5). These results indicate a high degree of nucleotide identity between the newly identified sequences and known bovine *Picornaviridae* strains, further supporting their taxonomic classification within their respective genera.

#### 3.3.6. *Circoviridae*

**Figure 6:**
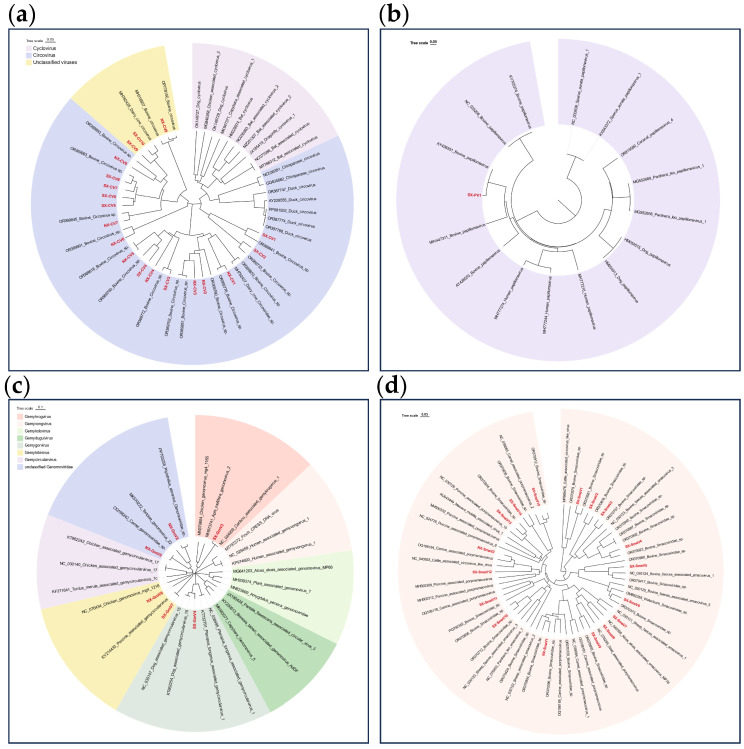
Phylogenetic analysis of relationships in DNA viruses: (**a**) Phylogenetic tree constructed based on the nucleotide sequences of the Rep gene of the family *Circoviridae*; (**b**) Phylogenetic tree based on the nucleotide sequences of the L1 gene of the family *Papillomaviridae*; (**c**) Phylogenetic tree built from the nucleotide sequences of the Rep gene of the family *Genomoviridae*; (**d**) Phylogenetic tree established using the nucleotide sequences of the Rep gene of the family *Smacoviridae*. Sequences highlighted in red represent viral sequences identified in this study.

Circoviruses are small, non-enveloped viruses characterized by a single-stranded circular DNA genome. The family *Circoviridae* comprises two genera: *Circovirus* and *Cyclovirus*. Members of the genus *Circovirus* infect vertebrates exclusively, whereas those of the genus *Cyclovirus* have been detected in both vertebrate and invertebrate hosts [37]. In this study, 19 Rep gene sequences of circoviruses (852–1094 bp in length) were obtained from DNA libraries in Shaanxi and Ningxia. Phylogenetic analysis revealed that all sequences clustered within the genus *Circovirus* (Figure 6a), forming a close evolutionary clade with reference strains of bovine-associated circoviruses from the Tibetan Plateau. Further sequence alignment demonstrated nucleotide identities of 79.31–99.23% in the Rep gene, with sequences distributed across distinct subclades. These results reveal significant genetic diversity of these viruses in bovine populations in western China.

#### 3.3.7. *Papillomaviridae*

*Papillomaviruses* are small, non-enveloped viruses with a circular double-stranded DNA genome that infect epithelial cells in a wide range of animal hosts, including humans, mammals, reptiles, and birds. These viruses have been extensively studied due to their association with proliferative lesions such as skin warts and papillomas [38]. In the present study, a nearly complete L1 gene nucleotide sequence (SX-PV1, 1529 bp in length) was obtained through sequencing. Phylogenetic analysis revealed that SX-PV1 exhibits 98.79% nucleotide sequence similarity to the L1 gene of *Bovine papillomavirus* from Japan (GenBank accession no. AY426551) and clusters within an unclassified branch of the family *Papillomaviridae* (Figure 6b).

#### 3.3.8. *Genomoviridae*

*Genomoviridae* is a widely distributed family of single-stranded DNA viruses [39]. In this study, six Rep gene nucleotide sequences (965–1128 bp in length) were assembled following sequencing. Phylogenetic analysis revealed that SX-GmV3 belongs to the genus *Gemykrogvirus*, clustering with *Caribou associated gemykrogvirus 1* from Canada (GenBank accession no. NC_024909) and sharing 99.69% nucleotide sequence identity (Figure 6c). SX-GmV4 was classified within the genus *Gemygorvirus* and exhibited 87.16% identity with *Faeces associated gemycircularvirus 15* detected in New Zealand (GenBank accession no. KT862254). NX-GmV6 and NX-GmV7 clustered within the *Gemykibivirus* branch, showing 81.33% and 99.47% identity with *Porcine feces-associated gemycircularvirus* and *Chicken genomovirus 4*, respectively. Furthermore, NX-GmV8 and NX-GmV9, which belong to an unclassified group within the family *Genomoviridae*, demonstrated 93% to 99% identity with two genomovirus sequences previously identified in herbivorous mammals from Northwest China.

#### 3.3.9. *Smacoviridae*

The family *Smacoviridae* comprises relatively novel single-stranded DNA viruses with genome sizes ranging from 2.3 to 2.9 kb. In this study, seven Rep gene nucleotide sequences (568–821 bp) were obtained from DNA libraries in Shaanxi and Ningxia provinces. Sequence alignment revealed that these sequences share high nucleotide similarity with bovine-associated smacoviruses, with identity levels of 83.62% to 94.34% for the Rep gene (Figure 6d). Among these, SX-SmaV1 was classified into the genus *Bonzesmacovirus*, exhibiting 83.62% nucleotide identity with *Bovine faeces-associated smacovirus 1*. The remaining sequences were identified as unclassified members of the family *Smacoviridae*.

## 4. Discussion

Bovine diseases not only severely impact the high-quality development of the dairy industry but also pose potential public health risks [40,41]. Although numerous metagenomic analyses have been conducted on various mammals, as well as vectors such as ticks and mosquitoes in Northwest China [42,43,44], characterization of the virome in dairy cattle from this region remains limited. Given the substantial scale of dairy farming in the Shaanxi–Gansu–Ningxia region, where milk production reached 5.422 million metric tons in 2022 (data from local statistical yearbooks), this study presents the first systematic investigation of viral diversity in dairy cattle from this area using metaviromics approaches. A total of 790 nasal and anal swab samples were collected from 13 sampling sites across the region to comprehensively elucidate the diversity of pathogenic agents and viral abundance within dairy cattle populations.

In the analysis of viral community diversity and abundance, a total of 51 viral families were identified, including major families such as *Astroviridae*, *Coronaviridae*, *Caliciviridae*, *Picornaviridae*, *Papillomaviridae*, *Circoviridae*, and *Genomoviridae*. Analysis revealed that among RNA viruses, *Astroviridae* was the most abundant in Ningxia, accounting for 47.20% of the total. *Picobirnaviridae* was predominant in Gansu, comprising 43.08%. In contrast, *Retroviridae* showed the highest abundance in Shaanxi at 64.41%. Notably, bovine leukemia virus (BLV) [45], a globally prevalent exogenous pathogenic retrovirus of dairy cattle with proven zoonotic potential, was not identified in any sequencing dataset in the present study. The dominant high-abundance *Retroviridae*-related reads detected in Shaanxi are speculated to predominantly originate from endogenous retroviruses (ERVs) stably integrated within the bovine host genome rather than infectious exogenous retroviruses [46]. The failure to capture BLV sequences is likely attributed to sample limitation: only nasal and anal swabs were collected in this survey, whereas BLV preferentially resides in peripheral blood lymphocytes and presents extremely low viral loads in mucosal swab specimens, leading to missed detection [47]. Considering the public health threat of BLV via raw dairy product exposure and close animal–human contact [48], further targeted epidemiological investigation using bovine blood samples combined with specific PCR and virus isolation is required to clarify the prevalence of exogenous pathogenic retroviruses and their underlying zoonotic risks across the Shaanxi–Gansu–Ningxia region. Among DNA viruses, phage sequences dominated the reads. Since bacteriophages are core constituents of the indigenous gastrointestinal virome of ruminants [49], and most samples were collected from free-range dairy farms under complex rearing conditions, prominent phage enrichment was observed, followed by CRESS-DNA viruses. *Siphoviridae* was the most common in both Gansu and Shaanxi, whereas *Microviridae* predominated in Ningxia. The α-diversity and richness of viruses, as indicated by Shannon and Richness indices, did not differ significantly (*p* > 0.05) among the three regions, which may be attributed to their geographical proximity, similar environmental conditions, and comparable human activity patterns. Located in Northwest China, the Shaanxi–Gansu–Ningxia region is an important livestock farming area. The three provinces are adjacent to each other and share geographical continuity, resulting in minimal differences in climate and ecological conditions. The agricultural and livestock production modes in these regions are highly similar. Frequent contact between cattle herds increases the likelihood of viral transmission across regions, explaining the lack of significant differences in viral diversity. Analysis of shared and unique viruses revealed seven common families and three common genera among RNA viruses. Notably, the unique presence of *Caliciviridae* (genus *Norovirus*) in Ningxia and *Betacoronavirus* in Gansu poses a potential risk of zoonotic transmission. For DNA viruses, nine common families and nine common genera were identified. Region-specific pathogens were detected, including *Herpesviridae* in Shaanxi, *Iridoviridae* in Gansu, and *Poxviridae* in Ningxia. Phylogenetic analysis demonstrated that the viruses carried by dairy cattle exhibit broad genetic diversity and some strains share high sequence similarity with viruses derived from heterologous hosts.

Coronaviruses are known to cause respiratory, enteric, and neurological diseases [50]. In this study, we identified a novel coronavirus sequence, designated NX-CoV1, which belongs to the genus *Betacoronavirus*. Viruses in this genus have historically caused severe human outbreaks through cross-species transmission, such as SARS-CoV and SARS-CoV-2 [31]. Sequence alignment analysis showed that NX-CoV1 shares 95.69% nucleotide identity with bovine coronavirus (BCoV) and 95.41% and 95.11% identity with two human coronavirus OC43 strains. Previous studies have shown that human coronavirus OC43, first isolated in 1967, shares up to 96% nucleotide identity with bovine coronavirus globally. Genetic analyses suggest that HCoV-OC43 likely crossed the species barrier from bovine coronavirus to humans and may be associated with the 1890 “Russian flu” pandemic [51]. Given the high sequence identity between NX-CoV1 and HCoV-OC43, NX-CoV1 may possess similar cross-species genetic characteristics, although this requires further experimental validation. The family *Picornaviridae* comprises 68 recognized genera, infecting a broad range of hosts and exhibiting diverse pathogenicity [52]. In this study, the sequenced reads were classified into four genera: *Enterovirus*, *Bopivirus*, *Aphthovirus*, and *Kobuvirus*. NX-PicV1, identified by sequencing, shares 92.95% nucleotide identity with *Bovine enterovirus E* (BEV-E) in the genus *Enterovirus*. This is consistent with other studies; for example, among 27 BEV strains isolated from major cattle-raising regions in China between 2012 and 2018, most were identified as EV-E, with only a few being EV-F [53]. Furthermore, studies have indicated that *Bovine enterovirus E* shares high genetic similarity with human enteroviruses in certain genomic regions, particularly in livestock environments, increasing the risk of cross-species transmission via contaminated water or food [54]. Additionally, NX-PicV3 exhibits 85.45% nucleotide identity with *Bovine rhinitis B virus* (GenBank No. KU168862) in the genus *Aphthovirus*. Other viruses in the genus *Aphthovirus*, such as foot-and-mouth disease virus (FMDV), are highly contagious in livestock, particularly ruminants. Although *Bovine rhinitis B* virus has low pathogenicity, its high sequence similarity suggests potential for cross-species transmission in high-density farming environments.

The family *Caliciviridae* represents a diverse group of viruses capable of infecting a wide range of hosts, including humans, pigs, cattle, cats, and dogs. Norovirus, a member of the genus *Norovirus* within this family, is a leading cause of viral gastroenteritis in humans worldwide. Based on the sequence of its major capsid protein (VP1), the norovirus genome can be classified into at least six genogroups (GI to GVI). Among these, most human infections and outbreak cases are primarily caused by genogroups GI and GII, whereas GIII predominantly infects ruminants, such as cattle and sheep. *Norovirus* GIII is further divided into two genotypes, GIII.1 and GIII.2. The viral sequence obtained in this study, designated NX-CaV1, clustered with *Norovirus* GIII.2. It has been reported that GIII.1 strains generally exhibit higher virulence than GIII.2 strains. Analysis of 211 diarrheal fecal samples collected from 25 farms across six provinces in China between 2017 and 2018 revealed that GIII.2 was the predominant genotype of bovine norovirus [55,56]. This finding suggests that although GIII.2 exhibits relatively lower virulence, its enhanced transmissibility within cattle populations may contribute to its status as the prevalent dominant strain. *Picobirnavirus* is a zoonotic pathogen with a broad host range, demonstrating significant potential for cross-species transmission among different hosts [57]. In this study, the newly identified sequences formed distinct phylogenetic clusters with strains of bovine picobirnavirus, camelus dromedarius picobirnavirus, porcine picobirnavirus, and marmota picobirnavirus.

Among DNA viruses, CRESS-DNA viruses represented the second most abundant group after bacteriophages. These viruses are circular single-stranded DNA viruses that encode a replication-associated protein (Rep) and primarily include members of the families *Circoviridae* and *Genomoviridae*. CRESS-DNA viruses have been associated with some cases of human diarrhea, potentially through the consumption of contaminated food products, such as pork and beef [58]. Within the family *Circoviridae*, 19 novel Rep gene sequences were identified. Phylogenetic analysis revealed that all these sequences clustered within the genus *Circovirus* and formed a tight evolutionary clade with bovine-associated Circovirus strains from the Qinghai–Tibet Plateau. The genomovirus sequences obtained in this study were distributed across three established genera and unclassified taxa within the family *Genomoviridae*. Among them, NX-GmV8 and NX-GmV9 exhibited 93% to 99% nucleotide identity with two previously reported *Genomoviridae* sequences from herbivorous mammals in Northwest China.

This study has several limitations. First, the samples did not comprehensively cover diverse geographical regions and environments; therefore, the generalizability of the findings requires further validation. Only clinically healthy dairy cows were sampled without a diseased control group. Most identified viruses were commensal or subclinical latent strains, whereas disease-causing viruses were poorly recovered, precluding any correlation between detected viruses and clinical disorders. Second, although we identified sequences highly similar to known human and animal coronaviruses, we lack experimental data to verify their actual capacity for cross-species transmission. Finally, this study primarily relied on sequence analysis and did not include functional or pathogenicity assessments. Future studies should further investigate the biological characteristics of these newly identified viruses and their potential implications for public health.

In conclusion, this study established a comprehensive reference viral database for dairy cattle in the Shaanxi–Gansu–Ningxia region through systematic metaviromic analysis, providing critical scientific basis for future monitoring, early warning, and control of viral diseases in this area. Furthermore, the viral genetic diversity and cross-species transmission potential uncovered in this study underscore the necessity for in-depth investigations into viral ecology and evolutionary mechanisms, thereby providing a theoretical foundation for sustainable development of the dairy industry and regional public health security.

## 5. Conclusions

In summary, this study presents the first systematic metaviromic analysis of viruses carried by dairy cattle in the Shaanxi–Gansu–Ningxia region, thereby filling the knowledge gap regarding the baseline viral diversity in this area. Using Illumina sequencing on 790 nasal and anal swab samples collected from dairy cattle between 2021 and 2023, this study identified and characterized 51 viral families, encompassing a wide range of RNA and DNA viruses. The results revealed no significant differences in viral diversity and abundance across different regions of the Shaanxi–Gansu–Ningxia area, while also demonstrating high genetic diversity and genetic characteristics related to cross-host transmission among these viruses. This study not only establishes a reference viral database for dairy cattle in the Shaanxi–Gansu–Ningxia region but also provides a critical scientific foundation for future viral surveillance and control strategies.

## Figures and Tables

**Table 1:** Overview of metavirome sequencing libraries for dairy cows.

Group	Sampling Year	Total Reads	Clean Reads	GC Content (%)	Classified Contigs	Viral Read Proportion (%)
SXN-RNA	2023	69,773,306	69,773,276	47.66	65,652	0.03
GSN-RNA	2023	73,017,650	73,017,634	46.96	49,893	0.13
NXN-RNA	2021–2022	52,401,848	51,775,112	55.41	62,817	0.19
SXN-DNA	2023	69,747,948	69,388,876	35.06	371,522	11.92
GSN-DNA	2023	69,294,684	69,294,598	43.25	314,489	5.11
NXN-DNA	2021–2022	74,000,446	73,510,804	40.41	152,072	2.10

## Data Availability

The raw data from the metavirome sequencing conducted in this study are available in the NCBI Sequence Read Archive (SRA) under BioProject accession PRJNA1180908.
